# Comparative genomic and phylogenetic analyses of the mitogenome of *Graptopetalum Paraguayense* (N. E. Br.) Walth. 1938

**DOI:** 10.1038/s41598-025-34236-4

**Published:** 2026-01-03

**Authors:** Xue Zhou, Qingming Ren, Chuqi Lin, Zhirui Li, Lin Zhou, Fei Xiong, Xi Du

**Affiliations:** 1 School of Chemistry and Biological Engineering, NanJing Normal University TaiZhou College, TaiZhou, 225300 China; 2 Fisheries College, Jimei University, Xiamen, 361021 China; 3 College of Biological Science and Technology of Yangzhou University, Yangzhou, 225009 China

**Keywords:** Graptopetalum paraguayense, Mitogenome, Phylogeny, Biotechnology, Genetics, Molecular biology, Plant sciences

## Abstract

*Graptopetalum paraguayense* is a perennial succulent plant with ornamental, ecological, and medicinal value. Here, we present the first complete mitogenome of *G. paraguayense*, assembled as a circular molecule of 242,059 bp with a GC content of 43.65%. The genome contains 50 genes, including 31 protein-coding genes (PCGs), 13 tRNAs, 3 rRNAs, and 3 pseudogenes. A total of 599 RNA editing sites were identified, with a predominant effect of altering amino acid hydrophobicity (47.41% were hydrophilic-to-hydrophobic conversions). Codon usage bias analysis revealed a preference for GCU (Ala), CGA (Arg), and UUA (Leu), with the stop codon UAA exhibiting the highest RSCU value (1.94). A total of 122 repetitive sequences were identified, comprising 59 simple sequence repeats (SSRs), 1 tandem repeat, and 62 dispersed repeats. Evolutionary analysis indicated positive selection on *ccmB* and *nad7* genes, while the majority of PCGs were under purifying selection. The mitogenome of *G. paraguayense* shared 57.28% sequence similarity with that of *Sedum plumbizincicola*. We also found evidence of chloroplast-to-mitochondrial DNA transfer, involving genes such as *psaC*, *ndhE*, *ndhG*, *ndhI*, *ndhA*, and *ndhH*. Comparative analyses identified eleven divergent hotspot regions: *atp9*, *atp8*, *rpl5*, *cox2*, *ccmFn*, *rps7*, *ccmC*, *ccmFc*, *mttB*, *nad6*, and *rps13*. Phylogenetic analysis confirmed the placement of *G. paraguayense* within the Acre clade of Crassulaceae, showing a sister relationship with *S. plumbizincicola*. Our study not only provides the first mitochondrial genomic resource for G. paraguayense but also reveals potential adaptive evolution through positive selection and interorganellar gene transfer, offering new perspectives on mitogenome plasticity in succulent plants.

**Supplementary Information:**

The online version contains supplementary material available at 10.1038/s41598-025-34236-4.

## Introduction

*Graptopetalum paraguayense (N. E. Br.)* Walth. 1938, a perennial succulent plant in the genus *Graptopetalum* of the Crassulaceae family, is commonly known as Ghost Plant or Mother of Pearl. Native to arid regions of Mexico, *G. paraguayense* features thick, flat leaves with slightly pointed tips, forming a lotus-like rosette that contributes to its high ornamental value. The leaf color varies with sunlight and environmental conditions: it appears blue-gray in the shade and pink under full sun. In addition to its ornamental value, *G. paraguayense* is also considered a functional food, valued for its antihypertensive and hepatoprotective properties. It has been reported to exhibit a range of other bioactive effects, including anticancer, antibacterial, antiasthma, skin-whitening, and anti-Alzheimer’s activities^1^. Moreover, *G. paraguayense* extract (GP extract) has been shown to inhibit liver cancer cell growth and significantly prolong the lifespan of *Caenorhabditis elegans*^2^.

The family Crassulaceae comprises 34 genera and over 1,500 species, including *Aeonium* and *Sedum*. Phylogenetically, Crassulaceae is divided into three subfamilies: Crassuloideae, Kalanchoideae, and Sempervivoideae. The Sempervivoideae subfamily is further divided into five major clades: Telephium, Sempervivum, Aeonium, Leucosedum, and Acre^3^. Previous studies have utilized various molecular markers, such as the nuclear *ETS*, internal transcribed spacer (*ITS*), and plastid DNA sequences (*rpl16*, *trnL-F*), to explore phylogenetic relationships within closely related genera of Crassulaceae (e.g., *Sedum* and *Echeveria*)^4^.

Beyond its medicinal and ornamental value, *G. paraguayense* serves as an important subject for evolutionary studies within Crassulaceae. Mitochondria are semi-autonomous organelles in eukaryotic cells. They play crucial roles in energy production, metabolism, and cellular signaling^5^. Plant mitogenomes are characterized by low mutation rates but frequent structural rearrangements, making them valuable markers for phylogenetic studies^6^. Plant mitochondria typically range from 1 to 3 μm in length and about 0.5 μm in diameter, with each cell containing several hundred of them^7^. The study of plant mitochondria began in earnest around the 1950 s, following the first successful isolations from animal and plant tissues^8^. Their structural features are complex and can include various conformations such as Y-type, H-type, single-ring, linear, and multi-ring structures. The mitogenome of *G. paraguayense*, the focus of this study, exhibits a ring-type configuration, highlighting the considerable variability in size and form across plant species^9^. The structure and function of mitochondria are intricately linked^10^, and their genomes exhibit considerable variability due to frequent recombination and horizontal gene transfer^11^. Owing to these characteristics, mitogenomes are invaluable tools for studying eukaryotic evolution, genetic diversity, species identification, and breeding new cultivars. The comparative analysis of mitochondrial DNA structure can also be used to trace common ancestry among different species^12^.

Ding et al. sequenced the mitogenome of *Sedum plumbizincicola* (Crassulaceae) using a combination of long-read (Oxford Nanopore Technology) and short-read (Illumina) sequencing platforms^6^. The assembled mitogenome was 212,159 bp in size with a GC content of 44.5%, containing 31 protein-coding genes (PCGs), 14 tRNAs, 3 rRNAs, 2 ORFs, and 11 pseudogenes. A notable finding was the identification of 508 RNA editing sites, 496 of which resulted in codon changes, predominantly nonsynonymous. This extensive RNA editing was proposed to significantly mitigate the impact of DNA mutations. Phylogenetic analysis positioned *S. plumbizincicola* as closely related to *Sedum album* within Crassulaceae^6^. Similarly, Yu et al. sequenced and compared the organelle genomes of three *Rhodiola* species (*R. wallichiana*, *R. crenulata*, and *R. sacra*) from the Qinghai-Tibet Plateau^13^. Their study revealed structural diversity among the mitogenomes, yet overall sequence evolution rates were slow. Phylogenetically, *R. wallichiana* and *R. sacra* were shown to be more closely related to each other than to *R. crenulata*^13^.

While significant progress has been made in studying the mitogenomes of Crassulaceae plants, research on *G. paraguayense* remains limited. Given their ecological significance, a comprehensive understanding of their mitogenomes is crucial for elucidating classification, phylogenetic relationships, and unique genetic characteristics. Furthermore, such studies would provide new insights into the evolutionary dynamics of Crassulaceae mitogenomes and could benefit broader plant genomic research and breeding programs. To fill this gap and provide evolutionary insights beyond descriptive genomics, we present the first complete mitogenome of *G. paraguayense*. Leveraging comparative and phylogenetic approaches, we aim to characterize its structural and compositional features, identify signals of selection and intergenomic transfer, and clarify its phylogenetic position within Crassulaceae.

## Materials and methods

### Plant samples and mitogenome sequencing

Fresh leaves of *Graptopetalum paraguayense* were collected from the Nanjing Ligan Flower Base in Nanjing, Jiangsu Province, China (32°12′N, 119°10′E). Healthy, fresh, and undamaged leaves were selected, thoroughly washed with sterile water, and stored at −80℃. Total genomic DNA was extracted using the CTAB method, and DNA quality was verified by spectrophotometry and agarose gel electrophoresis. The sequencing service was provided by Nanjing Genepioneer Biotechnologies (Nanjing, China) using PacBio Single-Molecule, Real-Time (SMRT) technology. Following DNA quality assessment, the samples were fragmented, and then subjected to end repair, adapter ligation, enzymatic digestion, and purification. Sequencing was performed on the PacBio Sequel II platform in HiFi mode. The resulting HiFi reads were quantified using a Perl script.

Genome assembly was conducted using PMAT2 software (v2.0.2) with the parameter “-g 5G”. The initial assembly results were visualized and manually adjusted using Bandage (v0.8.1). Finally, the HiFi reads were aligned back to the draft assembly using minimap2, and the sequence was polished with NextPolish to generate the final mitogenome sequence and its corresponding GFA file.

Protein-coding genes (PCGs) and ribosomal RNA genes (rRNA) were initially identified by aligning the sequence against published plant mitochondrial references using BLAST (v2.13.0). These initial annotations were manually refined based on information from closely related species. Transfer RNA genes (tRNAs) were predicted using tRNAscanSE (v2.0)^14^. Open reading frames (ORFs) were identified using the NCBI Open Reading Frame Finder with a minimum length of 102 bp, and redundant sequences or those overlapping known genes were excluded. Additionally, sequences longer than 300 bp that remained unannotated were subjected to BLAST analysis against the non-redundant (nr) protein database. RNA editing sites were predicted using PmtREP webserver^15^. All gene annotations were subsequently verified and manually curated. Finally, the circular mitogenome map was generated using OGDRAW (https://chlorobox.mpimp-golm.mpg.de/OGDraw.html).

### Relative synonymous codon usage (RSCU) analyses

The degeneracy of the genetic code allows most amino acids to be encoded by multiple synonymous codons. The unequal usage of these synonymous codons, known as Synonymous Codon Usage (RSCU), is a result of the combined effects of natural selection, mutation pressure, and genetic drift. To analyze codon usage bias, we calculated the RSCU values for all protein-coding genes. A custom Perl script (provided by Nanjing Genepioneer Biotechnologies, Nanjing, China) was used to extract unique coding sequences (CDS) for this analysis. The RSCU value for a codon is defined as the ratio of its observed frequency to the expected frequency if all synonymous codons for an amino acid were used equally. An RSCU value > 1 indicates that the codon is used more frequently than expected (positive bias), a value < 1 indicates less frequent use (negative bias), and a value = 1 indicates no usage bias.

### Repeat sequences analysis

Three types of repeat sequences were analyzed: simple sequence repeats (SSR), tandem repeats, and dispersed repeats. SSRs were identified using the MISA software (v1.0)^16^ with default parameters. Tandem repeats were detected using Tandem Repeats Finder (TRF, v4.09.1)^17^ with parameters set to 27 7 80 10 50 2000 -f -d -m. Dispersed repeats were identified by conducting an all-vs-all BLASTN (v2.10.1) alignment with the parameters: -word _ size 7, evalue 1e-5. The initial BLAST results were filtered to remove redundant hits and those corresponding to known tandem repeats. The genomic distribution of all repeat types was visualized using Circos (v0.69-5)^18^.

### Ka/Ks analyses

The ratio of nonsynonymous to synonymous substitution rates (Ka/Ks) was used to infer the type of selective pressure on protein-coding genes. Ka/Ks > 1 indicates positive selection, Ka/Ks < 1 indicates purifying selection, and Ka/Ks ≈ 1 suggests neutral evolution. For this analysis, the selected species were grouped into pairs, and homologous gene pairs were extracted between each pair. The coding sequences of these homologous pairs were aligned using MAFFT (v7.427)^19^. Subsequently, the Ka and Ks values for each alignment were calculated using KaKs_Calculator v2.0^20^ with the MLWL method. Finally, the Ka/Ks ratios for all gene pairs were compiled, and a box plot was generated to visualize the distribution of selection pressures across different species comparisons.

### Mitogenome synteny analysis

Mitogenome synteny was analyzed by performing whole-genome alignments against reference sequences using MUMmer (v4.0.0beta2) with the maxmatch parameter. The results were visualized as dot plots^21^.

### Nucleotide diversity and phylogenetic analysis

The complete mitogenomes of 14 species (Table S1) representing major clades within Crassulaceae and related saxifragalean families were retrieved from GenBank to provide a comprehensive phylogenetic context. Homologous gene sequences were aligned using MAFFT (v7.427) with the auto option. Nucleotide diversity (Pi values) was calculated for each gene alignment using DnaSP (v5)^19^.

For phylogenetic reconstruction, coding sequences (CDSs) were aligned with MAFFT, concatenated and then trimmed with trimAl (v1.4.rev15) using the –gt 0.7 parameter^22^. The best-fit substitution model (GTR) was selected using jModelTest (v2.1.10) under the Akaike Information Criterion (AIC). A maximum likelihood (ML) tree was inferred with RAxML (v8.2.10) under the GTRGAMMA model, with branch support assessed from 1,000 bootstrap replicates^23^.

### Migration analyses of chloroplast-to-mitochondrial DNA

Sequences homologous between the chloroplast and mitogenomes were identified using BLAST with an E-value cutoff of 1e–5 and a minimum sequence identity of 70%.

## Results

### Mitogenome assembly and annotation

**Fig. 1:**
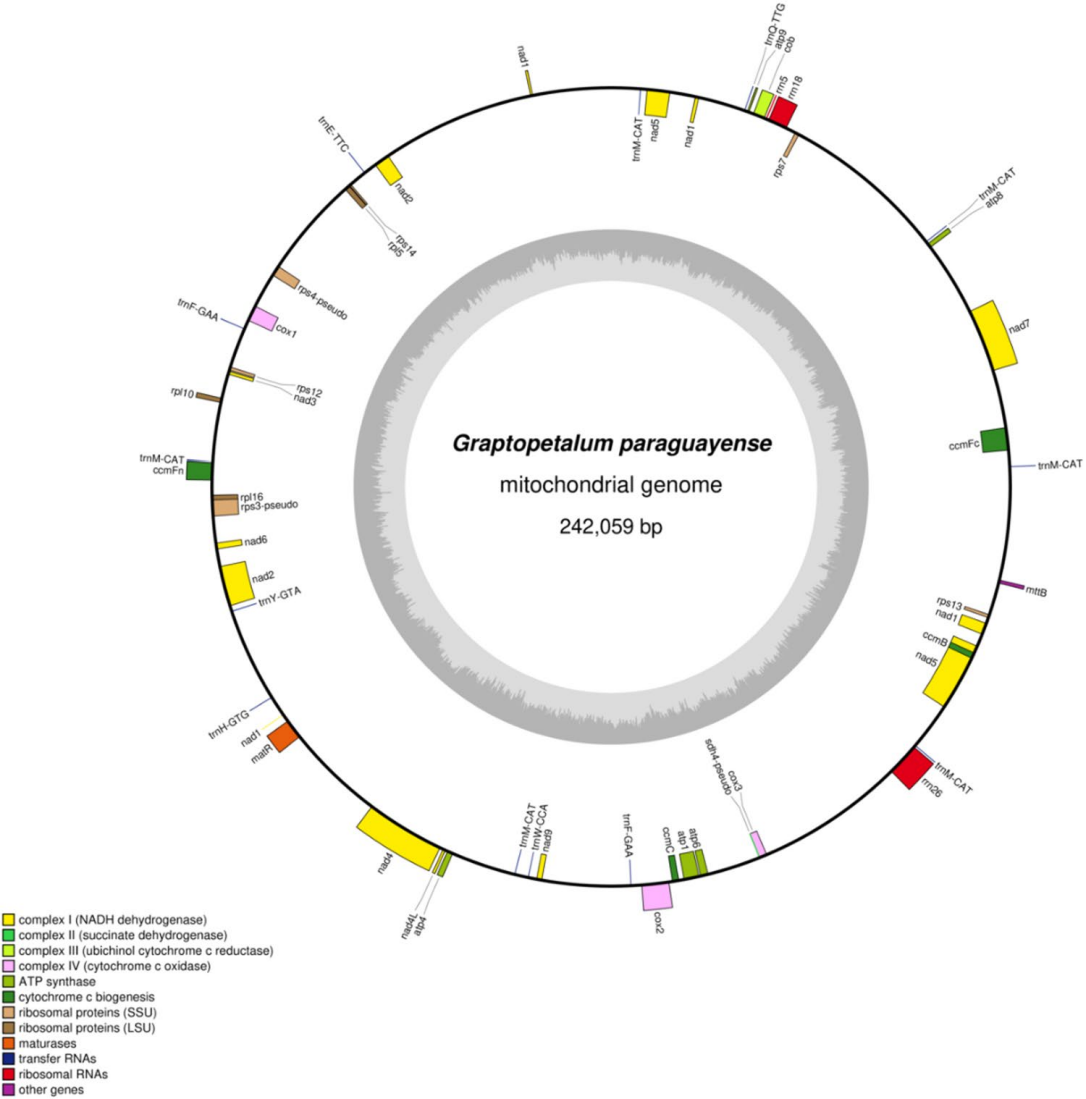
The complete mitochondria genomes of *G. paraguayense.*

The complete mitogenome sequence of *Graptopetalum paraguayense* was deposited in the NCBI database under accession number PV256627. The genome is 242,059 bp in length with a GC content of 43.65%. A total of 50 genes were annotated, comprising 31 protein-coding genes (PCGs), 13 transfer RNA (tRNA) genes, 3 ribosomal RNA (rRNA) genes, and 3 pseudogenes (Table 1, Table S2). A circular map of the genome was generated using OGDRAW (Fig. 1). Detailed assembly statistics are presented in Table S3. The completeness and gene content demonstrate a high-quality mitogenome assembly.

**Table 1:** The genes identified in the mitogenome of *G. paraguayense*.

Group of genes	Gene name
ATP synthase	*atp*1 *atp*4 *atp*6 *atp*8 *atp*9
Cytohrome c biogenesis	*ccm*B *ccm*C *ccm*Fc* *ccm*Fn*
Ubiquinol cytochrome c reductase	*cob*
Cytochrome c oxidase	*cox*1 *cox*2** *cox*3
Maturases	*mat*R
Transport membrane protein	*mtt*B
NADH dehydrogenase	*nad*1**** *nad*2**** *nad*3 *nad*4*** *nad*4L *nad*5**** *nad*6 *nad*7**** *nad*9
Ribosomal proteins (LSU)	*rpl*10 *rpl*16 *rpl*5
Ribosomal proteins (SSU)	#*rps*3 #*rps*4 *rps*12 *rps*13 *rps*14 *rps*7
Succinate dehydrogenase	#*sdh*4
Ribosomal RNAs	*rrn*18 *rrn*26 *rrn*5
Transfer RNAs	*trn*E-TTC *trn*F-GAA(2) *trn*H-GTG *trn*M-CAT(6) *trn*Q-TTG *trn*W-CCA *trn*Y-GTA

Note: *, number of introns; #, Pseudogene; (2), Number of copies for multi-copy genes.

Detailed gene annotations are provided in Table S2, assembly statistics in Table S3, codon usage data in Table S4, and repeat sequence analyses in Tables S5-S8.

**Fig. 2:**
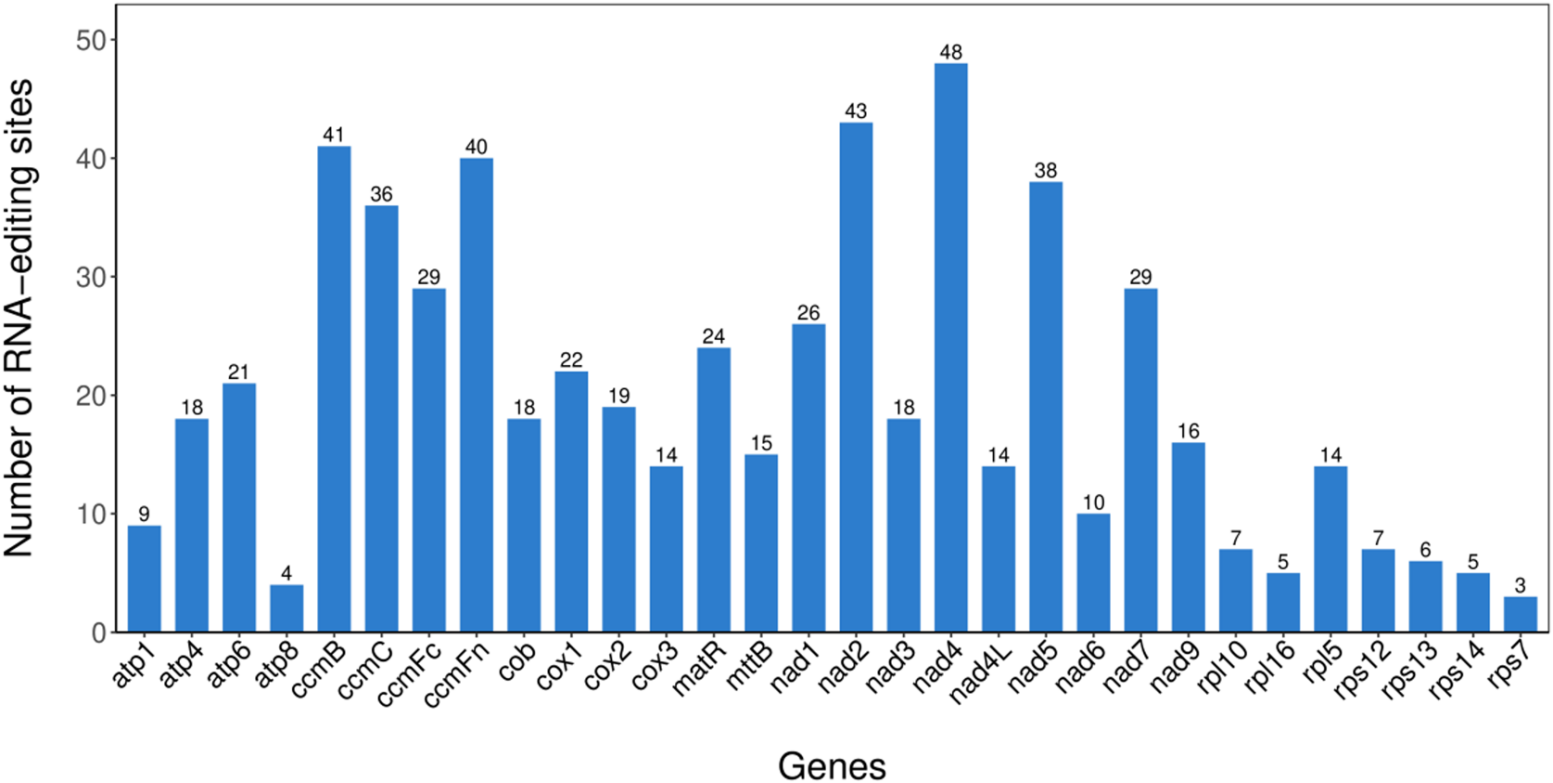
Statistics of the number of RNA editing sites for each gene.

RNA editing sites in the mitogenome were predicted through comparative analysis involving multiple sequence alignment. This process involved aligning the protein sequence of each gene with its homologs from related species and identifying codon positions where nucleotide substitutions would lead to changes in the encoded amino acid. Such sites were designated as predicted RNA editing site. Figure 2 presents the distribution of RNA editing sites across all protein-coding genes. A significant number of these editing events were found to alter the hydrophobicity or other physicochemical properties of the encoded amino acids (Fig. 2; Table 2), suggesting potential impacts on the structure and function of the resultant proteins.

**Table 2:** Effects of RNA editing on changes in hydrophilicity and hydrophobicity of amino acid in the mitogenome of *G. paraguayense*.

Type	RNA-editing	Number	Percentage
hydrophilic-hydrophilic	CAC (H) = > TAC (Y)	8	
	CAT (H) = > TAT (Y)	17	
	CGC (R) = > TGC (C)	11	
	CGT (R) = > TGT (C)	27	
	total	63	10.52%
hydrophilic-hydrophobic	ACA (T) = > ATA (I)	6	
	ACC (T) = > ATC (I)	6	
	ACG (T) = > ATG (M)	9	
	ACT (T) = > ATT (I)	7	
	CGG (R) = > TGG (W)	36	
	TCA (S) = > TTA (L)	78	
	TCC (S) = > TTC (F)	34	
	TCG (S) = > TTG (L)	48	
	TCT (S) = > TTT (F)	60	
	total	284	47.41%
	CAG (Q) = > TAG (X)	1	
	total	2	0.33%
hydrophobic-hydrophilic	CCA(P) = > TCA(S)	8	
	CCC (P) = > TCC (S)	17	
	CCG (P) = > TCG (S)	5	
	CCT (P) = > TCT (S)	19	
	total	49	8.18%
hydrophobic-hydrophobic	CCA (P) = > CTA (L)	45	
	CCC (P) = > CTC (L)	15	
	CCC (P) = > TTC (F)	4	
	CCG (P) = > CTG (L)	35	
	CCT (P) = > CTT (L)	27	
	CCT (P) = > TTT (F)	14	
	CTC (L) = > TTC (F)	15	
	CTT (L) = > TTT (F)	22	
	GCA (A) = > GTA (V)	8	
	GCC (A) = > GTC (V)	4	
	GCG (A) = > GTG (V)	7	
	GCT (A) = > GTT (V)	5	
	total	201	33.56%
	All	599	100%

### Codon usage bias analysis based on RSCU

The mitogenome of *G. paraguayense* utilized the standard 64 codons to encode 20 amino acids. The distribution of Relative Synonymous Codon Usage (RSCU) values showed that 30 codons were used more frequently than expected (RSCU > 1), 32 were used less frequently (RSCU < 1), and two codons—AUG (Met) and UGG (Trp)—showed no bias (RSCU = 1). The stop codon UAA exhibited the highest RSCU value (1.94), while the tyrosine codon UAC showed the strongest negative bias (RSCU = 0.44) (Table S4).

**Fig. 3:**
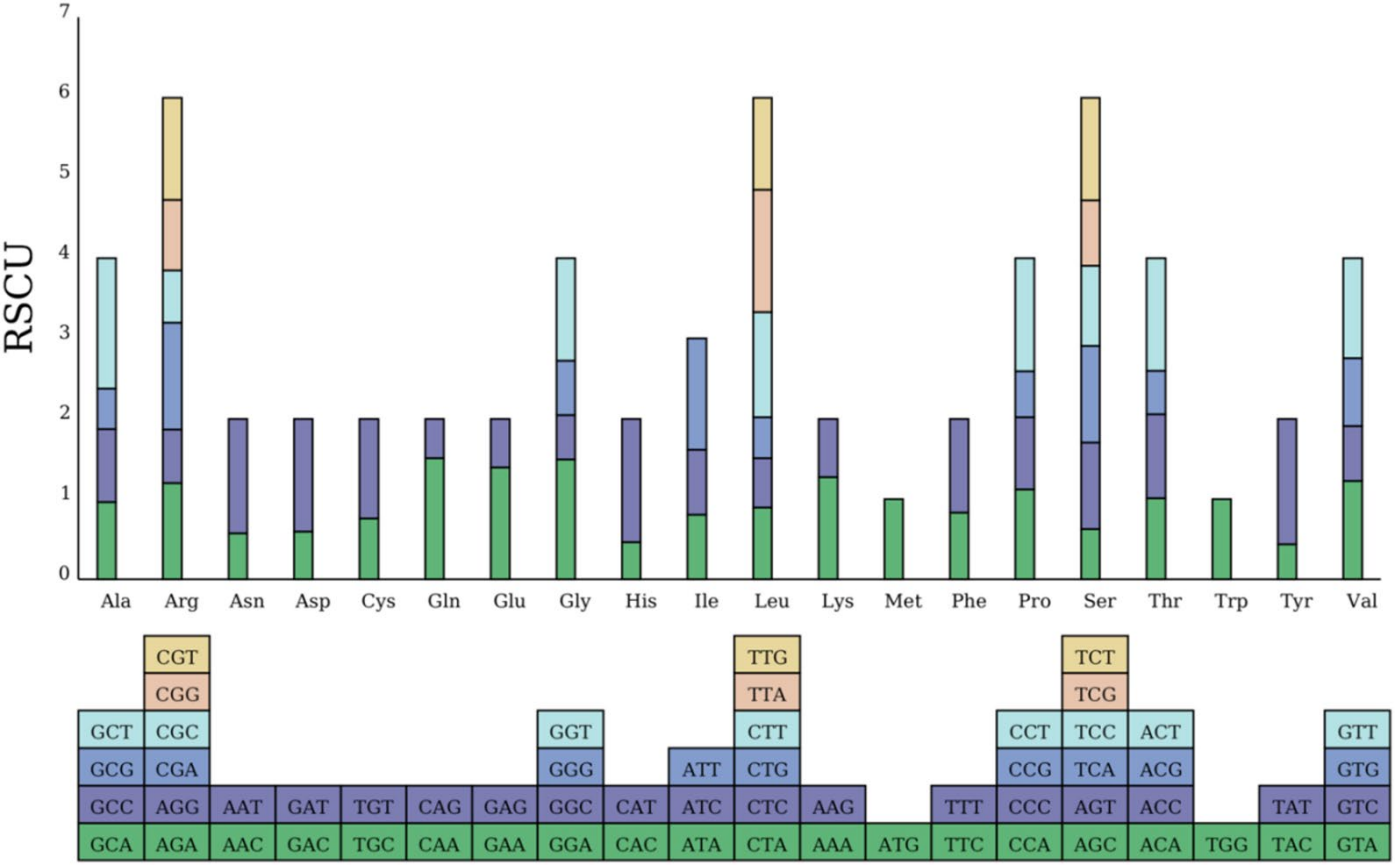
Relative Synonymous Codon Usage (RSCU)Value bar chart. The upper part of the picture shows the total RSCU of the mitochondria of the *G. paraguayense*, and the lower part represents the codons used.

Furthermore, for several amino acids, a clear preference for a specific synonymous codon was observed: GCU for alanine (Ala, RSCU = 1.62), CGA for arginine (Arg, RSCU = 1.33), GGA for glycine (Gly, RSCU = 1.50), UUA for leucine (Leu, RSCU = 1.52), and UCU for serine (Ser, RSCU = 1.28) (Fig. 3, Table S4).

### Analysis of repeat structures in the *G. paraguayense* mitogenome

**Fig. 4:**
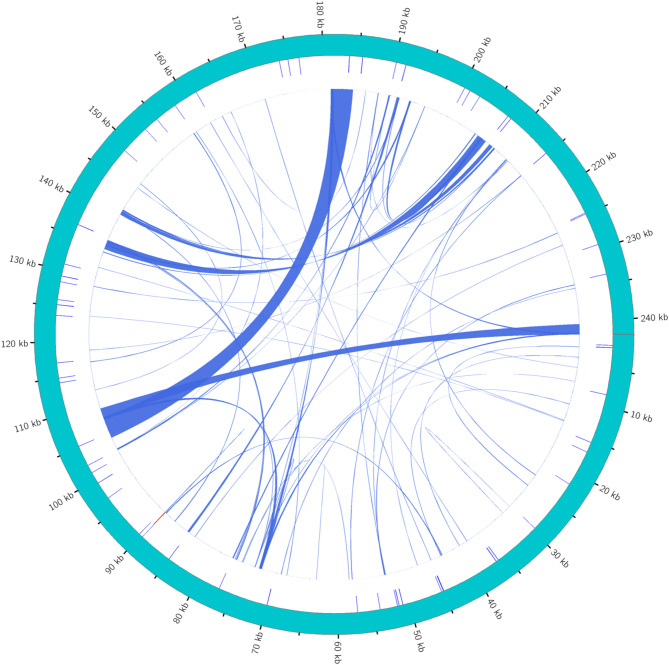
Distribution of repetitive sequences in the *G. paraguayense* mitogenome. The outermost circle shows the genome scale, followed by SSRs (green), tandem repeats (red), and dispersed repeats (blue) in successive circles.

Three types of repeat sequences were identified in the *G. paraguayense* mitogenome: simple sequence repeats (SSRs), tandem repeats, and dispersed repeats. In total, 122 repeat sequences were detected, consisting of 59 SSRs, one tandem repeat, and 62 dispersed repeats (Fig. 4, Table S5-S8).

### Analysis of evolutionary selection pressure using Ka/Ks ratios

**Fig. 5:**
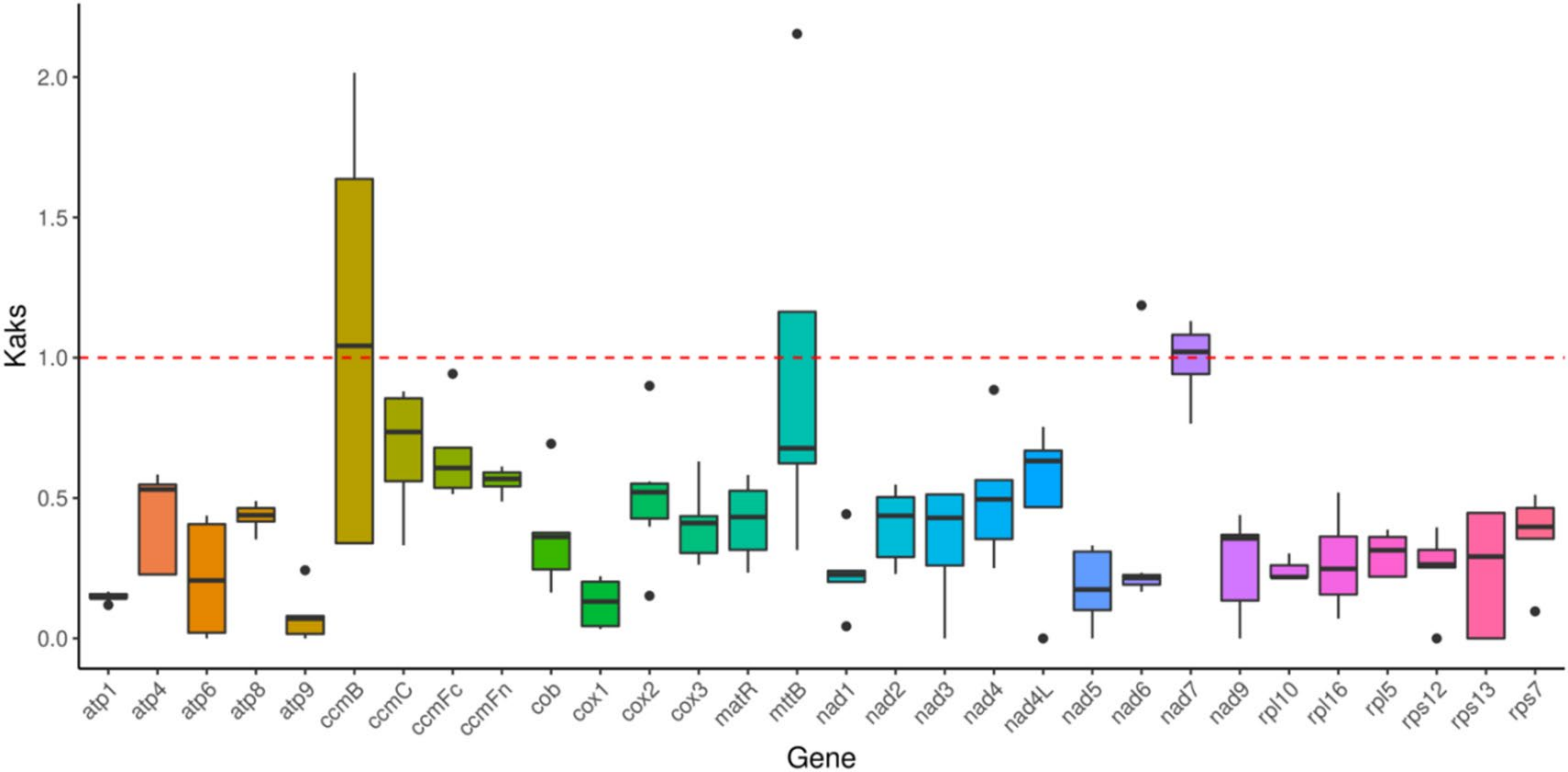
Species-based Ka/Ks bar line graph. The horizontal axis represents the gene name, and the vertical axis represents the ka/ks value. In the box plot, the upper and lower endpoints of the vertical lines above and below the rectangle respectively represent the upper and lower edges of the data, the thick line inside the rectangle represents the median, the upper and lower edges of the rectangle represent the upper and lower quartiles, and the dots outside the upper and lower edges of the data represent outliers.

To evaluate evolutionary constraints on mitochondrial protein-coding genes, we calculated Ka/Ks ratios for 30 genes shared between *G. paraguayense* and seven related species. Only *ccmB* and *nad7* showed median Ka/Ks values > 1 across species comparisons, indicating positive selection. The remaining 28 genes exhibited Ka/Ks < 1, consistent with widespread purifying selection that maintains functional integrity (Fig. 5). The analysis revealed that the median Ka/Ks ratio was greater than 1 for only two genes, *ccmB* and *nad7*, indicating that they have undergone positive selection. In contrast, the Ka/Ks ratios for the remaining 28 genes were all less than 1, consistent with widespread purifying selection. This group includes ribosomal protein genes (*rpl5*, *rpl10*, *rpl16*, *rps7*, *rps12*, *rps13*) and core respiratory complex genes (*cob*, *cox1-3*, *nad1-6*, *nad9*, *atp1-9*). No gene exhibited a Ka/Ks ratio equal to 1 (Fig. 5, Figure S9).

### Mitogenome synteny analysis

**Fig. 6:**
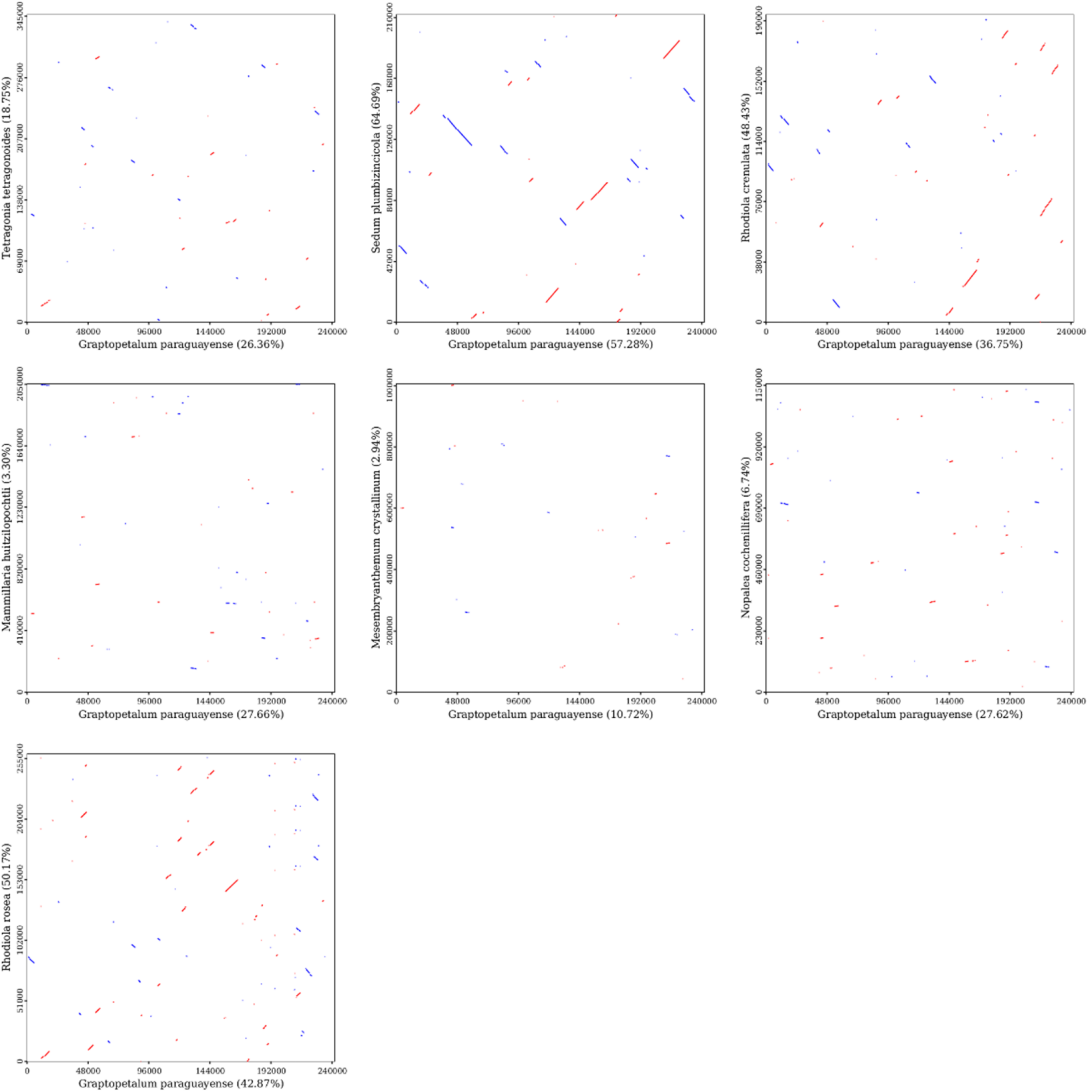
Collinear analysis of mitochondrial sequences. The horizontal axis in each box represents the assembled sequence, and the vertical axis represents other sequences. The values in parentheses indicate the proportion of homologous sequences in the total genome. The red lines in the boxes represent forward alignments, and the blue lines represent reverse complementary alignments.

Collinearity analysis was conducted by comparing the mitogenome of *G. paraguayense* with those of seven other plant species. Dot plot analysis revealed extensive collinearity between *G. paraguayense* and all compared species (Fig. 6). Specifically, *G. paraguayense* and *Sedum plumbizincicola* exhibited the highest degree of synteny, sharing long homologous sequences with 57.28% similarity. Conversely, the synteny with *Mesembryanthemum crystallinum* was the most limited, with homologous regions covering only 10.72% of the *G. paraguayense* mitogenome (Table S10).

### Nucleotide diversity and phylogenetic analysis

**Fig. 7:**
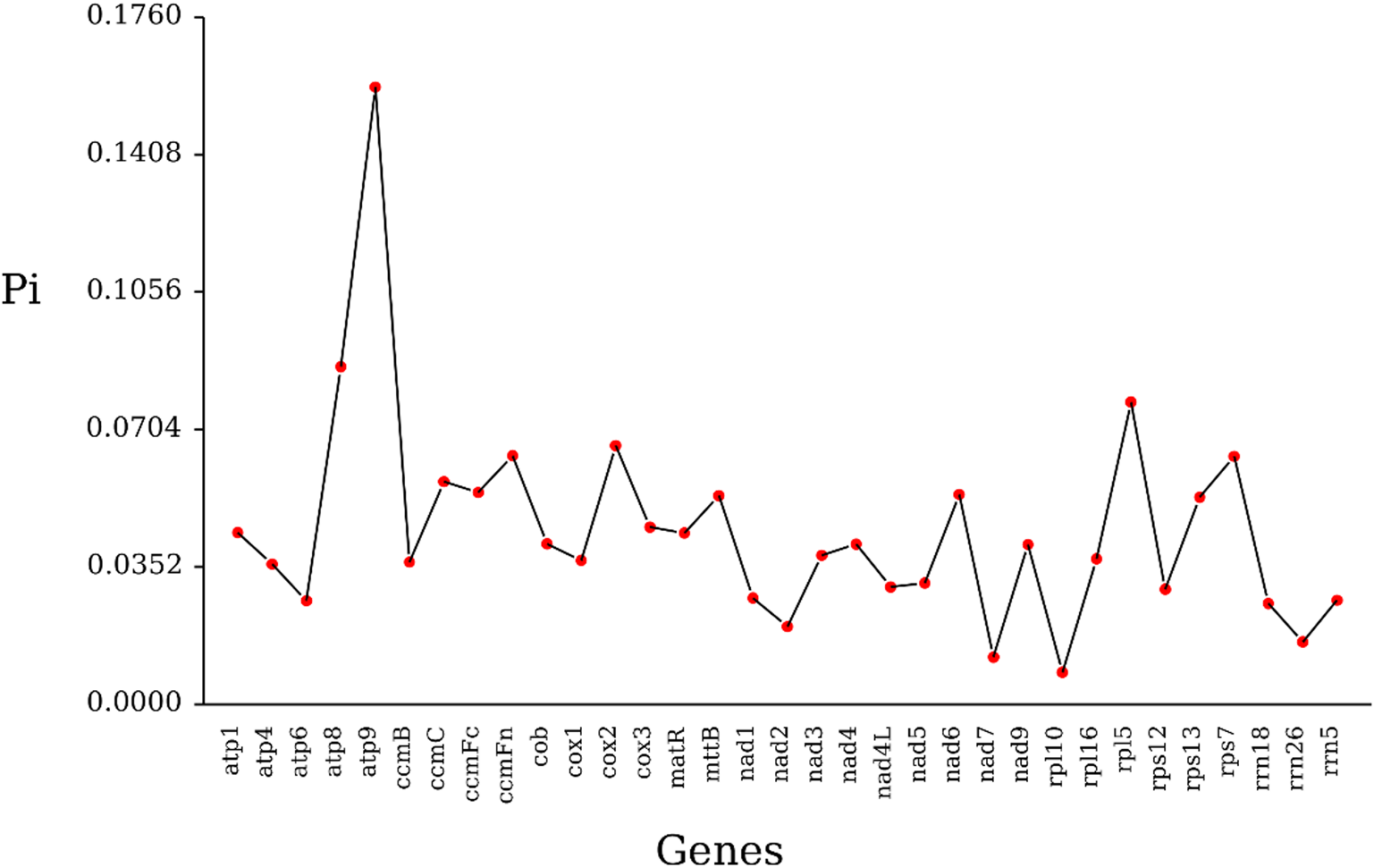
Gene Pi Value Line Chart. The x-axis represents gene names, and the y-axis indicates Pi values.

Analysis of nucleotide diversity (Pi) across the protein-coding genes (PCGs) of the *G.* paraguayense mitogenome revealed a wide range of variation. The *atp*9 gene exhibited the highest Pi value (0.15809), indicating the greatest sequence variability, followed by *atp*8 (Pi = 0.08645) and *rpl*5 (Pi = 0.07741). In contrast, genes such as *nad*7 (Pi = 0.01204) and *rrn*26 (Pi = 0.01596) showed notably lower variability. As expected, a positive correlation was observed between Pi values and the total number of variable sites. Genomic regions with high nucleotide diversity (Pi ≥ 0.05) are proposed as potential molecular markers for future population genetics or phylogenetic studies (Fig. 7, Table S11).

**Fig. 8:**
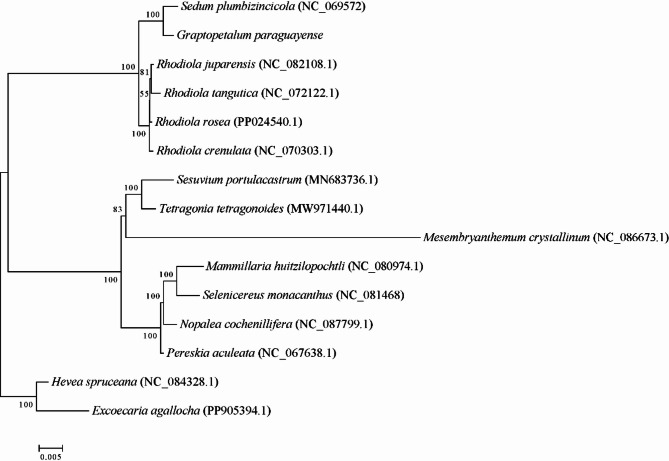
Analysis of mitochondrial system evolution. Evolutionary branch length: Also known as genetic variation or evolutionary distance. It represents the degree of change in evolutionary branches. The shorter it is, the smaller the difference and the closer the evolutionary distance. Distance scale: The unit length of the difference values between organisms or sequences, equivalent to the scale of an evolutionary tree. Bootstrap value: The bootstrap value is generally marked at the node position and is used to evaluate the credibility of the branch.

Maximum likelihood phylogenetic analysis strongly supported *G. paraguayense* and *S. plumbizincicola* as sister taxa (bootstrap support = 98%), confirming their close evolutionary relationship within the Acre clade. Species of *Rhodiola* formed a monophyletic clade, within which *R. crenulata* was more closely related to *R. rosea* (Fig. 8). This phylogenetic topology corroborates the taxonomic placement of *G. paraguayense* in the Acre clade and provides an evolutionary context for the high degree of mitochondrial sequence conservation (57.28% collinearity) observed between *G. paraguayense* and *S. plumbizincicola*.

### Identification of chloroplast-derived sequences in the mitogenome

BLAST analysis was performed to identify sequences homologous between the chloroplast and mitogenomes of *G. paraguayense*. The most significant hit (alignment score: 9,378) identified a ~ 5.7 kb homologous region. In the chloroplast genome, this region spanned coordinates 113,918–119,671 and contained the genes *psaC*, *ndhE*, *ndhG*, *ndhI*, *ndhA*, and *ndhH*. The homologous sequence in the mitogenome was located between coordinates 147,275 and 152,894 (Fig. 5). This finding provides clear evidence of a historical transfer of a large DNA fragment from the chloroplast to the mitogenome.

## Discussion

This study reports the first mitogenome of *G. paraguayense*, a species with both ornamental and medicinal value^1^. Beyond mere sequencing, our analyses reveal several evolutionary notable features: strong codon usage bias reflective of mutational pressure, evidence of chloroplast-to-mitochondrial DNA transfer, and signatures of positive selection on genes involved in cytochrome c biogenesis (*ccmB*) and NADH dehydrogenase assembly (*nad7*). These findings provide not only a genomic resource but also insights into mitogenome adaptation in arid-adapted succulents. Our analyses reveal several important features of its mitogenome architecture and evolutionary dynamics. Mitochondria are essential eukaryotic organelles enclosed by a double membrane and possessing their own genome and ribosomes^24,25^. They play critical roles not only in energy production but also in calcium storage and apoptosis regulation^26^. In this study, high-fidelity (HiFi) long-read sequencing of the *G. paraguayense* mitogenome was performed on the Pacific Biosciences Revio platform. Circular consensus sequencing (CCS) with stringent parameters (minimum passes ≥ 3, minimum read quality ≥ 0.99) and barcode demultiplexing (minimum score = 80) yielded 19,607 HiFi reads, representing 19.51 Gb (19,511,463,328 bp) of data. The mean read length was 20.55 kb with a median quality score of Q33. To assess potential exogenous contamination, 5,000 reads were randomly selected and aligned against the NT database using BLASTN. This quality control step revealed that 37.92% of the reads had no significant match to known species. Of the reads that had a significant match in the NT database, the most frequently identified species was *Graptopetalum amethystinum*. However, it constituted only 2.00% of the total read set, indicating a negligible level of contamination.

### Assembly and evolutionary conservation of the *G. paraguayense* mitogenome

The mitogenome of *G. paraguayense* was sequenced using PacBio Revio long-read technology and assembled into a single, circular molecule of 242,059 bp, demonstrating high continuity and completeness. Comparative genomic analysis placed its size within the typical range for angiosperms. Specifically, the *G. paraguayense* mitogenome is larger than those of its close relatives *Sedum plumbizincicola* (212,159 bp) and two *Rhodiola juparensis* (202,019 bp) and *Rhodiola crenulata* (194,106 bp). In contrast, it is substantially smaller than the mitogenomes of several other eudicots, including *Sesuvium portulacastrum* (392,221 bp), *Tetragonia tetragonoides* (347,227 bp), and the extremely large genomes of *Mesembryanthemum crystallinum* (1,005,707 bp). Its size is most comparable (differing by < 8%) to that of *Rhodiola rosea* (259,150 bp) and *R. tangutica* (257,378 bp).

Consistent with most plant mitogenomes (e.g., *Helianthus annuus* and *Sedum plumbizincicola*), the *rps2* gene is absent from the *G. paraguayense* mitogenome^27^. The *rps10* and *rps11* genes are also absent in *S. plumbizincicola* and several *Rhodiola* species (*R. juparensis*, *R. tangutica*, *R. rosea*, and *R. crenulate*), indicating an early evolutionary loss of these genes in the family. The *G. paraguayense* mitogenome encodes 13 tRNAs and 3 rRNAs, consistent with counts in related species: *S. plumbizincicola* (14 tRNAs, 3 rRNAs), *R. juparensis* (11 tRNAs, 3 rRNAs), and *R. tangutica* (15 tRNAs, 3 rRNAs). The overall GC content (43.65%) aligns with that of its relatives (ranging from 44.24% to 45.21%). Notably, the GC content of protein-coding regions (43.1%) was significantly lower than that of tRNA-coding (50.42%) and rRNA-coding regions (51.56%). Collectively, these findings highlight the evolutionary conservation of the *G. paraguayense* mitogenome. The conserved loss of *rps2*, *rps10*, and *rps11* genes across Crassulaceae suggests that these ribosomal protein genes were either functionally transferred to the nucleus or became dispensable early in the family’s evolution.

### Analysis of codon usage

Analysis of relative synonymous codon usage (RSCU) in the *G. paraguayense* mitogenome revealed significant bias. Of the 64 codons, 30 were preferentially used (RSCU > 1), 32 were underrepresented (RSCU < 1), and only two (AUG-Met and UGG-Trp) showed no bias (RSCU = 1). The stop codon UAA exhibited the strongest positive bias (RSCU = 1.94), while the tyrosine codon UAC showed the negative bias (RSCU = 0.44). For amino acids encoded by multiple synonymous codons, clear preferences were identified: GCU for alanine, CGA for arginine, GGA for glycine, UUA for leucine, and UCU for serine. The strong preference for A/U-ending codons (93.3% of preferred codons) correlates with the low overall GC content (43.65%), likely reflecting mutational bias toward AT-richness—a common feature in plant mitochondria that may influence translational efficiency and genome stability^28^. This bias is consistent with patterns observed in other Crassulaceae species, suggesting a conserved evolutionary trajectory in the family.

### Analysis of repetitive sequences and evolutionary selection pressure

We identified 122 repeat sequences in the *G. paraguayense* mitogenome, including 62 dispersed repeats that may facilitate homologous recombination and contribute to the structural plasticity observed in plant mitogenomes. The tandem repeat is 77 bp in length, spanning positions 89,779 to 89,855 (Fig. 4). The dispersed repeats ranged in size from 30 bp to 3,472 bp, and their detailed locations are provided in Table S5-S8.

To assess evolutionary selection pressure, we calculated Ka/Ks ratios for 30 protein-coding genes shared between *G. paraguayense* and seven related species (Table S9). The distribution of Ka/Ks values showed that only the *ccmB* and *nad7* genes had median values greater than 1, suggesting they are under positive selection (Fig. 5). The positive selection detected in *ccmB* (involved in cytochrome c maturation) and *nad7* (a subunit of respiratory complex I) suggests adaptive evolution of genes critical for mitochondrial electron transport and energy metabolism. This may reflect physiological adaptations to environmental stresses such as high light or drought, common in succulent habitats. In contrast, the remaining 28 genes had Ka/Ks ratios significantly less than 1, indicating widespread purifying selection and a high degree of functional conservation across the mitogenome^29^.

### Nucleotide diversity and phylogenetic relationships

Nucleotide diversity (Pi) analysis identified several highly variable genes (Pi ≥ 0.05) in the mitogenome, including *atp*9 (Pi = 0.158), *atp*8 (Pi = 0.086), *rpl*5 (Pi = 0.077), *cox*2 (Pi = 0.066), *ccm*Fn (Pi = 0.064), *rps*7 (Pi = 0.063), *ccm*C (Pi = 0.057), *ccm*Fc (Pi = 0.054), *mtt*B (Pi = 0.053), *nad*6 (Pi = 0.054), and *rps*13 (Pi = 0.053). These genes exhibited a high number of variant sites (e.g., 66 in *atp*9 and 194 in *ccm*Fn), indicating accelerated evolutionary rates and substantial genetic polymorphisms^30^. These hypervariable regions therefore represent promising molecular markers for future phylogenetic, population genetic, and adaptive evolutionary studies within closely related species^31^.

The phylogenetic reconstruction strongly supported *G. paraguayense* and *S. plumbizincicola* as sister taxa, unequivocally placing *G. paraguayense* within the Acre clade. Species of the genus *Rhodiola* formed a monophyletic group, with *R. crenulata* and *R. rosea* being closely related. This robust phylogenetic framework provides essential evolutionary context for interpreting the patterns of nucleotide diversity observed in *G. paraguayense*.

## Conclusions

This study presents the first complete mitogenome of *G. paraguayense*, providing fundamental genomic resources for Crassulaceae. Key findings include: (1) a 242,059 bp circular genome with 50 genes and abundant RNA editing sites; (2) strong codon usage bias favoring A/U-ending codons; (3) positive selection on ccmB and nad7 genes; (4) identification of highly variable regions suitable as molecular markers; (5) evidence of chloroplast-to-mitochondrial DNA transfer; and (6) robust phylogenetic placement within the Acre clade. Moreover, our analyses highlight adaptive signatures in respiratory genes, identify highly variable regions suitable for phylogenetic markers, and document chloroplast-derived DNA transfer. These findings pave the way for future studies on organellar evolution, stress adaptation, and phylogenetic reconstruction in succulent plants.

## Supplementary Information

Below is the link to the electronic supplementary material.

Supplementary Material 1

## Data Availability

The data is available at NCBI accession: PV256627.
