# Supplementary material for: Comparative genomic and phylogenetic analyses of the mitogenome of Graptopetalum Paraguayense (N. E. Br.) Walth. 1938
Source: Sci Rep. 2026 Jan 3;16:4174. doi: 10.1038/s41598-025-34236-4 (PMC12859013; doi:10.1038/s41598-025-34236-4)
Supplement: Supplementary file 1 — Supplementary Material 1 [file 41598_2025_34236_MOESM1_ESM.docx]

Comparative genomic and phylogenetic analyses of the mitogenome of *Graptopetalum paraguayense* (N. E. Br.) Walth. 1938

Xue Zhou^a^, Qingming Ren^c^, Chuqi Lin^a, b^, Zhirui Li^a^, Lin Zhou^a^, Fei Xiong^c^, Xi Du^a^ *

^a^ School of Chemistry and Biological Engineering, NanJing Normal University TaiZhou College, TaiZhou 225300, China

^b^ Fisheries College, Jimei University, Xiamen 361021, China

^c^ College of Biological Science and Technology of Yangzhou University, Yangzhou 225009, China

Table S1. The complete mitogenomes of 14 species downloaded from GenBank.

Table S2. Mitochondrial genome-encoded genes in *G. paraguayense*

Table S3. Summary of sequence data generated for whole mitochondrial genome sequencing of *G. paraguayense*.

Table S4. Codon usage and RSCU values in the mitogenomes of *G. paraguayense*.

Table S5. *G. paraguayense* dispersed repeats of the mitochondrial genome.

Table S6. *G. paraguayense* simple sequence repeat of the mitochondrial genome.

Table S7. *G. paraguayense* tandem repeats of the mitochondrial genome.

Table S8. The amount and type of repeated sequences in the mitogenomes of *G. paraguayense*.

Table S8. The amount and type of repeated sequences in the mitogenomes of *G. paraguayense*.

Table S9. The Ka/Ks ratio for each gene pair.

Table S10. Mitochondrial Sequence Collinearity Analysis Results.

Table S11. The Pi value of each gene.

Table S1. The complete mitogenomes of 14 species downloaded from GenBank.

| Species name | GenBank Accession No. |
| --- | --- |
| *Sedum plumbizincicola* | NC_069572 |
| *Rhodiola juparensis* | NC_082108.1 |
| *Rhodiola tangutica* | NC_072122.1 |
| *Rhodiola rose* | PP024540.1 |
| *Rhodiola crenulate* | NC_070303.1 |
| *Sesuvium portulacastrum* | MN683736.1 |
| *Tetragonia tetragonoides* | MW971440.1 |
| *Mesembryanthemum crystallinum* | NC_086673.1 |
| *Mammillaria huitzilopochtli* | NC_080974.1 |
| *Selenicereus monacanthus* | NC_081468 |
| *Nopalea cochenillifera* | NC_087799.1 |
| *Pereskia aculeata* | NC_067638.1 |
| *Hevea spruceana* | NC_084328.1 |
| *Excoecaria agallocha* | PP905394.1 |

Table S2. Mitochondrial genome-encoded genes in *G. paraguayense*

| Species | Gene | tRNA | rRNA | mRNA | Pseudo |
| --- | --- | --- | --- | --- | --- |
| *Graptopetalum paraguayense* | 50 | 13 | 3 | 31 | 3 |

Table S3. Summary of sequence data generated for whole mitochondrial genome sequencing of *G. paraguayense*.

| Species | Number of Reads | Number of Bases | Mean Read Length | N50 Read Length |
| --- | --- | --- | --- | --- |
| *Graptopetalum paraguayense* | 949437 | 19547174233 | 20588 | 20433 |

Table S4. Codon usage and RSCU values in the mitogenomes of *G. paraguayense*.

| AminoAcid | Symbol | Codon | No. | RSCU |
| --- | --- | --- | --- | --- |
| * | Ter | UAA | 20 | 1.9355 |
| A | Ala | GCU | 240 | 1.6244 |
| Y | Tyr | UAU | 214 | 1.562 |
| H | His | CAU | 168 | 1.5342 |
| L | Leu | UUA | 236 | 1.5226 |
| Q | Gln | CAA | 188 | 1.51 |
| G | Gly | GGA | 239 | 1.4961 |
| N | Asn | AAU | 190 | 1.4232 |
| P | Pro | CCU | 183 | 1.405 |
| T | Thr | ACU | 157 | 1.4049 |
| D | Asp | GAU | 203 | 1.4048 |
| E | Glu | GAA | 242 | 1.3948 |
| I | Ile | AUU | 327 | 1.3876 |
| R | Arg | CGA | 121 | 1.3297 |
| L | Leu | CUU | 203 | 1.3097 |
| S | Ser | UCU | 176 | 1.28 |
| G | Gly | GGU | 204 | 1.277 |
| K | Lys | AAA | 213 | 1.2754 |
| R | Arg | CGU | 116 | 1.2747 |
| V | Val | GUU | 174 | 1.2429 |
| C | Cys | UGU | 80 | 1.2403 |
| V | Val | GUA | 172 | 1.2286 |
| S | Ser | UCA | 166 | 1.2073 |
| R | Arg | AGA | 109 | 1.1978 |
| F | Phe | UUU | 330 | 1.1702 |
| L | Leu | UUG | 178 | 1.1484 |
| P | Pro | CCA | 146 | 1.1209 |
| S | Ser | AGU | 148 | 1.0764 |
| T | Thr | ACC | 117 | 1.047 |
| T | Thr | ACA | 113 | 1.0112 |
| M | Met | AUG | 243 | 1 |
| W | Trp | UGG | 132 | 1 |
| S | Ser | UCC | 137 | 0.9964 |
| A | Ala | GCA | 142 | 0.9611 |
| A | Ala | GCC | 135 | 0.9137 |
| P | Pro | CCC | 117 | 0.8983 |
| L | Leu | CUA | 139 | 0.8968 |
| R | Arg | CGG | 80 | 0.8791 |
| V | Val | GUG | 119 | 0.85 |
| F | Phe | UUC | 234 | 0.8298 |
| S | Ser | UCG | 112 | 0.8145 |
| I | Ile | AUA | 190 | 0.8062 |
| I | Ile | AUC | 190 | 0.8062 |
| C | Cys | UGC | 49 | 0.7597 |
| K | Lys | AAG | 121 | 0.7246 |
| V | Val | GUC | 95 | 0.6786 |
| G | Gly | GGG | 108 | 0.6761 |
| R | Arg | AGG | 61 | 0.6703 |
| R | Arg | CGC | 59 | 0.6484 |
| S | Ser | AGC | 86 | 0.6255 |
| L | Leu | CUC | 95 | 0.6129 |
| E | Glu | GAG | 105 | 0.6052 |
| D | Asp | GAC | 86 | 0.5952 |
| * | Ter | UGA | 6 | 0.5806 |
| N | Asn | AAC | 77 | 0.5768 |
| P | Pro | CCG | 75 | 0.5758 |
| G | Gly | GGC | 88 | 0.5509 |
| T | Thr | ACG | 60 | 0.5369 |
| L | Leu | CUG | 79 | 0.5097 |
| A | Ala | GCG | 74 | 0.5008 |
| Q | Gln | CAG | 61 | 0.49 |
| * | Ter | UAG | 5 | 0.4839 |
| H | His | CAC | 51 | 0.4658 |
| Y | Tyr | UAC | 60 | 0.438 |

Note: Amino acids are represented by single-letter abbreviations, "*" denotes a stop codon, and "No." indicates the count of that codon.

Table S5. *G. paraguayense* dispersed repeats of the mitochondrial genome.

| #Chr1 | Chr2 | type | alignment length | | similarity | | start1 | | end1 | start2 | end2 | evalue |
| --- | --- | --- | --- | --- | --- | --- | --- | --- | --- | --- | --- | --- |
| chr1 | chr1 | P | 30 | 100 | | 164805 | | 164834 | | 102377 | 102406 | 5.67e-07 |
| chr1 | chr1 | F | 31 | 96.774 | | 31322 | | 31352 | | 29880 | 29910 | 7.33e-06 |
| chr1 | chr1 | F | 32 | 96.875 | | 211457 | | 211488 | | 47147 | 47178 | 2.04e-06 |
| chr1 | chr1 | F | 34 | 97.059 | | 186478 | | 186511 | | 163276 | 163309 | 1.58e-07 |
| chr1 | chr1 | P | 35 | 100 | | 13718 | | 13752 | | 131711 | 131745 | 9.42e-10 |
| chr1 | chr1 | P | 35 | 97.143 | | 75200 | | 75234 | | 58154 | 58188 | 4.38e-08 |
| chr1 | chr1 | P | 36 | 94.444 | | 211768 | | 211803 | | 188516 | 188550 | 2.04e-06 |
| chr1 | chr1 | F | 38 | 94.737 | | 145678 | | 145715 | | 5159 | 5196 | 4.38e-08 |
| chr1 | chr1 | F | 38 | 92.105 | | 89818 | | 89855 | | 89779 | 89816 | 2.04e-06 |
| chr1 | chr1 | P | 39 | 92.308 | | 75341 | | 75379 | | 7393 | 7431 | 5.67e-07 |
| chr1 | chr1 | P | 41 | 92.683 | | 58154 | | 58194 | | 75194 | 75234 | 4.38e-08 |
| chr1 | chr1 | F | 44 | 95.455 | | 196090 | | 196131 | | 102213 | 102256 | 7.28e-11 |
| chr1 | chr1 | F | 44 | 95.455 | | 196090 | | 196131 | | 190206 | 190249 | 7.28e-11 |
| chr1 | chr1 | P | 47 | 95.745 | | 234738 | | 234783 | | 68894 | 68940 | 1.57e-12 |
| chr1 | chr1 | P | 47 | 93.617 | | 123899 | | 123945 | | 13834 | 13880 | 2.02e-11 |
| chr1 | chr1 | P | 48 | 97.917 | | 170505 | | 170552 | | 44872 | 44919 | 2.60e-15 |
| chr1 | chr1 | F | 48 | 95.833 | | 161139 | | 161184 | | 38475 | 38522 | 4.35e-13 |
| chr1 | chr1 | P | 48 | 91.667 | | 131711 | | 131758 | | 13707 | 13752 | 9.42e-10 |
| chr1 | chr1 | F | 49 | 100 | | 227690 | | 227738 | | 83732 | 83780 | 1.55e-17 |
| chr1 | chr1 | P | 49 | 89.796 | | 63306 | | 63353 | | 63306 | 63353 | 1.22e-08 |
| chr1 | chr1 | P | 50 | 96 | | 211754 | | 211803 | | 52414 | 52462 | 3.36e-14 |
| chr1 | chr1 | F | 52 | 86.538 | | 158500 | | 158550 | | 67996 | 68047 | 5.67e-07 |
| chr1 | chr1 | F | 56 | 83.929 | | 146460 | | 146515 | | 112253 | 112306 | 7.33e-06 |
| chr1 | chr1 | F | 57 | 92.982 | | 73147 | | 73202 | | 72111 | 72167 | 9.35e-15 |
| chr1 | chr1 | P | 57 | 92.982 | | 107739 | | 107795 | | 73147 | 73202 | 9.35e-15 |
| chr1 | chr1 | F | 57 | 92.982 | | 181014 | | 181070 | | 73147 | 73202 | 9.35e-15 |
| chr1 | chr1 | F | 57 | 92.982 | | 241824 | | 241880 | | 73147 | 73202 | 9.35e-15 |
| chr1 | chr1 | P | 58 | 82.759 | | 76501 | | 76558 | | 135787 | 135844 | 7.33e-06 |
| chr1 | chr1 | F | 59 | 88.136 | | 214898 | | 214956 | | 118553 | 118611 | 2.02e-11 |
| chr1 | chr1 | F | 59 | 83.051 | | 43162 | | 43220 | | 11034 | 11091 | 7.33e-06 |
| chr1 | chr1 | P | 60 | 91.667 | | 73147 | | 73205 | | 107736 | 107795 | 9.35e-15 |
| chr1 | chr1 | P | 65 | 92.308 | | 39863 | | 39927 | | 3538 | 3601 | 1.55e-17 |
| chr1 | chr1 | P | 67 | 80.597 | | 135787 | | 135853 | | 76492 | 76558 | 7.33e-06 |
| chr1 | chr1 | F | 72 | 81.944 | | 208945 | | 209016 | | 191828 | 191898 | 4.38e-08 |
| chr1 | chr1 | F | 74 | 98.649 | | 188477 | | 188550 | | 52375 | 52448 | 9.16e-30 |
| chr1 | chr1 | F | 75 | 93.333 | | 25443 | | 25517 | | 1909 | 1983 | 1.19e-23 |
| chr1 | chr1 | P | 75 | 86.667 | | 214890 | | 214962 | | 57637 | 57711 | 9.35e-15 |
| chr1 | chr1 | P | 82 | 86.585 | | 141069 | | 141150 | | 87709 | 87790 | 1.55e-17 |
| chr1 | chr1 | F | 87 | 97.701 | | 225590 | | 225675 | | 128585 | 128671 | 9.09e-35 |
| chr1 | chr1 | F | 87 | 95.402 | | 191836 | | 191921 | | 141498 | 141583 | 1.97e-31 |
| chr1 | chr1 | P | 88 | 85.227 | | 87709 | | 87796 | | 141063 | 141150 | 1.55e-17 |
| chr1 | chr1 | F | 90 | 77.778 | | 211526 | | 211612 | | 23439 | 23527 | 7.33e-06 |
| chr1 | chr1 | F | 95 | 77.895 | | 234165 | | 234258 | | 52506 | 52598 | 5.67e-07 |
| chr1 | chr1 | P | 109 | 100 | | 158014 | | 158122 | | 116695 | 116803 | 6.88e-51 |
| chr1 | chr1 | P | 116 | 75.862 | | 136058 | | 136172 | | 76216 | 76330 | 1.58e-07 |
| chr1 | chr1 | P | 130 | 80 | | 89563 | | 89692 | | 42931 | 43056 | 4.32e-18 |
| chr1 | chr1 | F | 133 | 75.188 | | 205084 | | 205215 | | 134189 | 134314 | 5.67e-07 |
| chr1 | chr1 | P | 171 | 74.854 | | 206230 | | 206400 | | 76828 | 76998 | 1.21e-13 |
| chr1 | chr1 | P | 182 | 72.527 | | 76216 | | 76396 | | 135992 | 136172 | 1.58e-07 |
| chr1 | chr1 | F | 204 | 99.51 | | 190182 | | 190385 | | 102189 | 102392 | 4.96e-102 |
| chr1 | chr1 | F | 215 | 76.279 | | 193583 | | 193795 | | 135337 | 135549 | 1.19e-23 |
| chr1 | chr1 | F | 216 | 77.315 | | 208802 | | 209016 | | 141352 | 141560 | 1.98e-26 |
| chr1 | chr1 | F | 244 | 100 | | 241816 | | 242059 | | 72103 | 72346 | 6.19e-126 |
| chr1 | chr1 | F | 244 | 100 | | 241816 | | 242059 | | 181006 | 181249 | 6.19e-126 |
| chr1 | chr1 | F | 279 | 74.194 | | 206201 | | 206475 | | 193537 | 193811 | 4.29e-23 |
| chr1 | chr1 | F | 324 | 99.074 | | 191515 | | 191838 | | 84891 | 85213 | 7.57e-165 |
| chr1 | chr1 | P | 425 | 99.765 | | 107380 | | 107803 | | 72103 | 72527 | 0 |
| chr1 | chr1 | F | 425 | 99.765 | | 181006 | | 181429 | | 72103 | 72527 | 0 |
| chr1 | chr1 | F | 607 | 73.147 | | 207943 | | 208533 | | 140697 | 141292 | 4.14e-48 |
| chr1 | chr1 | F | 1553 | 73.02 | | 205654 | | 207180 | | 134741 | 136265 | 7.84e-140 |
| chr1 | chr1 | P | 1655 | 99.94 | | 240406 | | 242059 | | 107560 | 109214 | 0 |
| chr1 | chr1 | P | 3472 | 100 | | 181006 | | 184477 | | 104332 | 107803 | 0 |

Table S6. *G. paraguayense* simple sequence repeat of the mitochondrial genome.

| ID | SSR nr. | SSR type | SSR | size | start | end |
| --- | --- | --- | --- | --- | --- | --- |
| chr1 | 1 | p4 | (CGAT)3 | 12 | 1495 | 1506 |
| chr1 | 2 | p1 | (C)10 | 10 | 1831 | 1840 |
| chr1 | 3 | p4 | (GGAC)3 | 12 | 8388 | 8399 |
| chr1 | 4 | p1 | (G)11 | 11 | 15314 | 15324 |
| chr1 | 5 | p2 | (TA)5 | 10 | 16756 | 16765 |
| chr1 | 6 | p4 | (AGAA)3 | 12 | 21820 | 21831 |
| chr1 | 7 | p4 | (AATG)3 | 12 | 29572 | 29583 |
| chr1 | 8 | p2 | (AT)5 | 10 | 36251 | 36260 |
| chr1 | 9 | p1 | (T)10 | 10 | 36595 | 36604 |
| chr1 | 10 | p1 | (C)11 | 11 | 44940 | 44950 |
| chr1 | 11 | p4 | (GGCG)3 | 12 | 44985 | 44996 |
| chr1 | 12 | p2 | (AT)5 | 10 | 45265 | 45274 |
| chr1 | 13 | p4 | (AGAA)3 | 12 | 50900 | 50911 |
| chr1 | 14 | p1 | (A)13 | 13 | 51433 | 51445 |
| chr1 | 15 | p2 | (AT)5 | 10 | 51675 | 51684 |
| chr1 | 16 | p4 | (CGGC)3 | 12 | 54128 | 54139 |
| chr1 | 17 | p1 | (A)10 | 10 | 57265 | 57274 |
| chr1 | 18 | p1 | (T)10 | 10 | 69906 | 69915 |
| chr1 | 19 | p4 | (GTAT)3 | 12 | 76922 | 76933 |
| chr1 | 20 | p4 | (GCAG)3 | 12 | 84962 | 84973 |
| chr1 | 21 | p1 | (A)10 | 10 | 89768 | 89777 |
| chr1 | 22 | p4 | (CAAT)3 | 12 | 90320 | 90331 |
| chr1 | 23 | p1 | (T)17 | 17 | 96912 | 96928 |
| chr1 | 24 | p3 | (TTC)4 | 12 | 99398 | 99409 |
| chr1 | 25 | p4 | (AGCT)3 | 12 | 101079 | 101090 |
| chr1 | 26 | p1 | (A)10 | 10 | 102226 | 102235 |
| chr1 | 27 | p1 | (T)10 | 10 | 105145 | 105154 |
| chr1 | 28 | p1 | (A)11 | 11 | 114314 | 114324 |
| chr1 | 29 | p3 | (GTA)4 | 12 | 114972 | 114983 |
| chr1 | 30 | p1 | (T)13 | 13 | 117090 | 117102 |
| chr1 | 31 | p4 | (AATG)3 | 12 | 123671 | 123682 |
| chr1 | 32 | p1 | (A)10 | 10 | 125151 | 125160 |
| chr1 | 33 | p1 | (T)11 | 11 | 125859 | 125869 |
| chr1 | 34 | p3 | (AGC)4 | 12 | 128361 | 128372 |
| chr1 | 35 | p3 | (AAT)4 | 12 | 129179 | 129190 |
| chr1 | 36 | p1 | (G)10 | 10 | 130863 | 130872 |
| chr1 | 37 | p3 | (ACA)4 | 12 | 136634 | 136645 |
| chr1 | 38 | p4 | (TATT)3 | 12 | 148774 | 148785 |
| chr1 | 39 | p1 | (A)10 | 10 | 152872 | 152881 |
| chr1 | 40 | p4 | (GAAG)3 | 12 | 154972 | 154983 |
| chr1 | 41 | p2 | (AG)8 | 16 | 158150 | 158165 |
| chr1 | 42 | p1 | (G)10 | 10 | 161592 | 161601 |
| chr1 | 43 | p4 | (CTTA)3 | 12 | 173898 | 173909 |
| chr1 | 44 | p4 | (CTTT)3 | 12 | 175107 | 175118 |
| chr1 | 45 | p4 | (TACT)3 | 12 | 176646 | 176657 |
| chr1 | 46 | p1 | (A)10 | 10 | 183655 | 183664 |
| chr1 | 47 | p2 | (TA)6 | 12 | 185492 | 185503 |
| chr1 | 48 | p1 | (A)10 | 10 | 190219 | 190228 |
| chr1 | 49 | p4 | (GCAG)3 | 12 | 191586 | 191597 |
| chr1 | 50 | p1 | (T)15 | 15 | 200310 | 200324 |
| chr1 | 51 | p2 | (CT)5 | 10 | 201139 | 201148 |
| chr1 | 52 | p2 | (CT)5 | 10 | 202704 | 202713 |
| chr1 | 53 | p6 | (GAATAA)3 | 18 | 207454 | 207471 |
| chr1 | 54 | p2 | (AT)5 | 10 | 207988 | 207997 |
| chr1 | 55 | p4 | (GAGT)3 | 12 | 214799 | 214810 |
| chr1 | 56 | p1 | (A)10 | 10 | 224697 | 224706 |
| chr1 | 57 | p1 | (T)10 | 10 | 224833 | 224842 |
| chr1 | 58 | p1 | (T)10 | 10 | 229405 | 229414 |
| chr1 | 59 | p4 | (CATA)3 | 12 | 233642 | 233653 |

Table S7. *G. paraguayense* tandem repeats of the mitochondrial genome.

| NO. | Chr | Size | Copy | Repeat sequence | Percent *Mat*ches | Start | End |
| --- | --- | --- | --- | --- | --- | --- | --- |
| 1 | chr1 | 39 | 2 | AACAGCTACGGACAGCTAGAAACAAACTCTTATATCAGA | 92 | 89779 | 89855 |

Table S8. The amount and type of repeated sequences in the mitogenomes of *G. paraguayense*.

| Species | Simple sequence repeat | | Tandem repeat | | Dispersed repeat | Total |
| --- | --- | --- | --- | --- | --- | --- |
| *Graptopetalum_paraguayense* | | 59 | 1 | 62 | | 122 |

Table S9. The Ka/Ks ratio for each gene pair.

| Kaks | Gene | Vs |
| --- | --- | --- |
| 0.144206 | *atp*1 | Graptopetalum_paraguayense vs MW971440.1 |
| 0.119037 | *atp*1 | Graptopetalum_paraguayense vs NC_069572 |
| 0.161488 | *atp*1 | Graptopetalum_paraguayense vs NC_070303.1 |
| 0.143692 | *atp*1 | Graptopetalum_paraguayense vs NC_080974.1 |
| NA | *atp*1 | Graptopetalum_paraguayense vs NC_086673.1 |
| 0.145543 | *atp*1 | Graptopetalum_paraguayense vs NC_087799.1 |
| 0.166358 | *atp*1 | Graptopetalum_paraguayense vs PP024540.1 |
| 0.53069 | *atp*4 | Graptopetalum_paraguayense vs MW971440.1 |
| NA | *atp*4 | Graptopetalum_paraguayense vs NC_069572 |
| 0.227766 | *atp*4 | Graptopetalum_paraguayense vs NC_070303.1 |
| 0.583606 | *atp*4 | Graptopetalum_paraguayense vs NC_080974.1 |
| NA | *atp*4 | Graptopetalum_paraguayense vs NC_086673.1 |
| 0.547972 | *atp*4 | Graptopetalum_paraguayense vs NC_087799.1 |
| 0.226332 | *atp*4 | Graptopetalum_paraguayense vs PP024540.1 |
| 0.437431 | *atp*6 | Graptopetalum_paraguayense vs MW971440.1 |
| 0.0803458 | *atp*6 | Graptopetalum_paraguayense vs NC_069572 |
| 0 | *atp*6 | Graptopetalum_paraguayense vs NC_070303.1 |
| 0.43177 | *atp*6 | Graptopetalum_paraguayense vs NC_080974.1 |
| NA | *atp*6 | Graptopetalum_paraguayense vs NC_086673.1 |
| 0.332162 | *atp*6 | Graptopetalum_paraguayense vs NC_087799.1 |
| 0 | *atp*6 | Graptopetalum_paraguayense vs PP024540.1 |
| 0.352774 | *atp*8 | Graptopetalum_paraguayense vs MW971440.1 |
| 0.402614 | *atp*8 | Graptopetalum_paraguayense vs NC_069572 |
| 0.464239 | *atp*8 | Graptopetalum_paraguayense vs NC_070303.1 |
| 0.489355 | *atp*8 | Graptopetalum_paraguayense vs NC_080974.1 |
| 0.438891 | *atp*8 | Graptopetalum_paraguayense vs NC_086673.1 |
| 0.430438 | *atp*8 | Graptopetalum_paraguayense vs NC_087799.1 |
| 0.464239 | *atp*8 | Graptopetalum_paraguayense vs PP024540.1 |
| 0.0631166 | *atp*9 | Graptopetalum_paraguayense vs MW971440.1 |
| 0 | *atp*9 | Graptopetalum_paraguayense vs NC_069572 |
| NA | *atp*9 | Graptopetalum_paraguayense vs NC_070303.1 |
| 0.0800642 | *atp*9 | Graptopetalum_paraguayense vs NC_080974.1 |
| 0.242993 | *atp*9 | Graptopetalum_paraguayense vs NC_086673.1 |
| 0.0779512 | *atp*9 | Graptopetalum_paraguayense vs NC_087799.1 |
| 0 | *atp*9 | Graptopetalum_paraguayense vs PP024540.1 |
| 1.79253 | *ccm*B | Graptopetalum_paraguayense vs MW971440.1 |
| 0.33816 | *ccm*B | Graptopetalum_paraguayense vs NC_069572 |
| 0.338939 | *ccm*B | Graptopetalum_paraguayense vs NC_070303.1 |
| 2.01499 | *ccm*B | Graptopetalum_paraguayense vs NC_080974.1 |
| 1.04235 | *ccm*B | Graptopetalum_paraguayense vs NC_086673.1 |
| 1.48123 | *ccm*B | Graptopetalum_paraguayense vs NC_087799.1 |
| 0.338939 | *ccm*B | Graptopetalum_paraguayense vs PP024540.1 |
| 0.866641 | *ccm*C | Graptopetalum_paraguayense vs MW971440.1 |
| NA | *ccm*C | Graptopetalum_paraguayense vs NC_069572 |
| 0.332061 | *ccm*C | Graptopetalum_paraguayense vs NC_070303.1 |
| 0.820688 | *ccm*C | Graptopetalum_paraguayense vs NC_080974.1 |
| 0.650386 | *ccm*C | Graptopetalum_paraguayense vs NC_086673.1 |
| 0.879882 | *ccm*C | Graptopetalum_paraguayense vs NC_087799.1 |
| 0.529921 | *ccm*C | Graptopetalum_paraguayense vs PP024540.1 |
| 0.514353 | *ccm*Fc | Graptopetalum_paraguayense vs MW971440.1 |
| 0.679979 | *ccm*Fc | Graptopetalum_paraguayense vs NC_069572 |
| 0.942793 | *ccm*Fc | Graptopetalum_paraguayense vs NC_070303.1 |
| 0.536656 | *ccm*Fc | Graptopetalum_paraguayense vs NC_080974.1 |
| NA | *ccm*Fc | Graptopetalum_paraguayense vs NC_086673.1 |
| 0.535983 | *ccm*Fc | Graptopetalum_paraguayense vs NC_087799.1 |
| 0.677484 | *ccm*Fc | Graptopetalum_paraguayense vs PP024540.1 |
| 0.612286 | *ccm*Fn | Graptopetalum_paraguayense vs MW971440.1 |
| 0.48775 | *ccm*Fn | Graptopetalum_paraguayense vs NC_069572 |
| NA | *ccm*Fn | Graptopetalum_paraguayense vs NC_070303.1 |
| 0.541666 | *ccm*Fn | Graptopetalum_paraguayense vs NC_080974.1 |
| NA | *ccm*Fn | Graptopetalum_paraguayense vs NC_086673.1 |
| 0.591344 | *ccm*Fn | Graptopetalum_paraguayense vs NC_087799.1 |
| 0.56851 | *ccm*Fn | Graptopetalum_paraguayense vs PP024540.1 |
| 0.379946 | *cob* | Graptopetalum_paraguayense vs MW971440.1 |
| 0.693619 | *cob* | Graptopetalum_paraguayense vs NC_069572 |
| NA | *cob* | Graptopetalum_paraguayense vs NC_070303.1 |
| 0.358452 | *cob* | Graptopetalum_paraguayense vs NC_080974.1 |
| 0.208127 | *cob* | Graptopetalum_paraguayense vs NC_086673.1 |
| 0.365255 | *cob* | Graptopetalum_paraguayense vs NC_087799.1 |
| 0.163653 | *cob* | Graptopetalum_paraguayense vs PP024540.1 |
| 0.188464 | *cox*1 | Graptopetalum_paraguayense vs MW971440.1 |
| 0.0738908 | *cox*1 | Graptopetalum_paraguayense vs NC_069572 |
| 0.0338198 | *cox*1 | Graptopetalum_paraguayense vs NC_070303.1 |
| 0.221246 | *cox*1 | Graptopetalum_paraguayense vs NC_080974.1 |
| NA | *cox*1 | Graptopetalum_paraguayense vs NC_086673.1 |
| 0.206191 | *cox*1 | Graptopetalum_paraguayense vs NC_087799.1 |
| 0.0338198 | *cox*1 | Graptopetalum_paraguayense vs PP024540.1 |
| 0.528229 | *cox*2 | Graptopetalum_paraguayense vs MW971440.1 |
| 0.151829 | *cox*2 | Graptopetalum_paraguayense vs NC_069572 |
| NA | *cox*2 | Graptopetalum_paraguayense vs NC_070303.1 |
| 0.513264 | *cox*2 | Graptopetalum_paraguayense vs NC_080974.1 |
| 0.559105 | *cox*2 | Graptopetalum_paraguayense vs NC_086673.1 |
| 0.398273 | *cox*2 | Graptopetalum_paraguayense vs NC_087799.1 |
| 0.899882 | *cox*2 | Graptopetalum_paraguayense vs PP024540.1 |
| 0.435925 | *cox*3 | Graptopetalum_paraguayense vs MW971440.1 |
| 0.630347 | *cox*3 | Graptopetalum_paraguayense vs NC_069572 |
| 0.281731 | *cox*3 | Graptopetalum_paraguayense vs NC_070303.1 |
| 0.410861 | *cox*3 | Graptopetalum_paraguayense vs NC_080974.1 |
| 0.262839 | *cox*3 | Graptopetalum_paraguayense vs NC_086673.1 |
| 0.435305 | *cox*3 | Graptopetalum_paraguayense vs NC_087799.1 |
| 0.327051 | *cox*3 | Graptopetalum_paraguayense vs PP024540.1 |
| 0.45987 | *mat*R | Graptopetalum_paraguayense vs MW971440.1 |
| 0.404826 | *mat*R | Graptopetalum_paraguayense vs NC_069572 |
| 0.285576 | *mat*R | Graptopetalum_paraguayense vs NC_070303.1 |
| 0.582135 | *mat*R | Graptopetalum_paraguayense vs NC_080974.1 |
| NA | *mat*R | Graptopetalum_paraguayense vs NC_086673.1 |
| 0.548016 | *mat*R | Graptopetalum_paraguayense vs NC_087799.1 |
| 0.234405 | *mat*R | Graptopetalum_paraguayense vs PP024540.1 |
| 0.58926 | *mtt*B | Graptopetalum_paraguayense vs MW971440.1 |
| 0.31479 | *mtt*B | Graptopetalum_paraguayense vs NC_069572 |
| 1.16365 | *mtt*B | Graptopetalum_paraguayense vs NC_070303.1 |
| 0.677349 | *mtt*B | Graptopetalum_paraguayense vs NC_080974.1 |
| 2.15424 | *mtt*B | Graptopetalum_paraguayense vs NC_086673.1 |
| 0.658312 | *mtt*B | Graptopetalum_paraguayense vs NC_087799.1 |
| 1.16365 | *mtt*B | Graptopetalum_paraguayense vs PP024540.1 |
| 0.20134 | *nad*1 | Graptopetalum_paraguayense vs MW971440.1 |
| 0.442816 | *nad*1 | Graptopetalum_paraguayense vs NC_069572 |
| NA | *nad*1 | Graptopetalum_paraguayense vs NC_070303.1 |
| 0.22683 | *nad*1 | Graptopetalum_paraguayense vs NC_080974.1 |
| NA | *nad*1 | Graptopetalum_paraguayense vs NC_086673.1 |
| 0.240603 | *nad*1 | Graptopetalum_paraguayense vs NC_087799.1 |
| 0.0432498 | *nad*1 | Graptopetalum_paraguayense vs PP024540.1 |
| 0.436949 | *nad*2 | Graptopetalum_paraguayense vs MW971440.1 |
| 0.229549 | *nad*2 | Graptopetalum_paraguayense vs NC_069572 |
| 0.349438 | *nad*2 | Graptopetalum_paraguayense vs NC_070303.1 |
| 0.490149 | *nad*2 | Graptopetalum_paraguayense vs NC_080974.1 |
| 0.516837 | *nad*2 | Graptopetalum_paraguayense vs NC_086673.1 |
| 0.54768 | *nad*2 | Graptopetalum_paraguayense vs NC_087799.1 |
| 0.229963 | *nad*2 | Graptopetalum_paraguayense vs PP024540.1 |
| 0.346164 | *nad*3 | Graptopetalum_paraguayense vs MW971440.1 |
| NA | *nad*3 | Graptopetalum_paraguayense vs NC_069572 |
| NA | *nad*3 | Graptopetalum_paraguayense vs NC_070303.1 |
| 0.512711 | *nad*3 | Graptopetalum_paraguayense vs NC_080974.1 |
| NA | *nad*3 | Graptopetalum_paraguayense vs NC_086673.1 |
| 0.512711 | *nad*3 | Graptopetalum_paraguayense vs NC_087799.1 |
| 0 | *nad*3 | Graptopetalum_paraguayense vs PP024540.1 |
| 0.495625 | *nad*4 | Graptopetalum_paraguayense vs MW971440.1 |
| 0.885565 | *nad*4 | Graptopetalum_paraguayense vs NC_069572 |
| 0.313981 | *nad*4 | Graptopetalum_paraguayense vs NC_070303.1 |
| 0.564151 | *nad*4 | Graptopetalum_paraguayense vs NC_080974.1 |
| 0.250501 | *nad*4 | Graptopetalum_paraguayense vs NC_086673.1 |
| 0.563826 | *nad*4 | Graptopetalum_paraguayense vs NC_087799.1 |
| 0.39351 | *nad*4 | Graptopetalum_paraguayense vs PP024540.1 |
| 0.75336 | *nad*4L | Graptopetalum_paraguayense vs MW971440.1 |
| 0 | *nad*4L | Graptopetalum_paraguayense vs NC_069572 |
| NA | *nad*4L | Graptopetalum_paraguayense vs NC_070303.1 |
| 0.641055 | *nad*4L | Graptopetalum_paraguayense vs NC_080974.1 |
| NA | *nad*4L | Graptopetalum_paraguayense vs NC_086673.1 |
| 0.623228 | *nad*4L | Graptopetalum_paraguayense vs NC_087799.1 |
| NA | *nad*4L | Graptopetalum_paraguayense vs PP024540.1 |
| 0.331132 | *nad*5 | Graptopetalum_paraguayense vs MW971440.1 |
| 0 | *nad*5 | Graptopetalum_paraguayense vs NC_069572 |
| 0.174069 | *nad*5 | Graptopetalum_paraguayense vs NC_070303.1 |
| 0.319742 | *nad*5 | Graptopetalum_paraguayense vs NC_080974.1 |
| 0.156527 | *nad*5 | Graptopetalum_paraguayense vs NC_086673.1 |
| 0.298586 | *nad*5 | Graptopetalum_paraguayense vs NC_087799.1 |
| 0.0450418 | *nad*5 | Graptopetalum_paraguayense vs PP024540.1 |
| 0.190542 | *nad*6 | Graptopetalum_paraguayense vs MW971440.1 |
| 1.18672 | *nad*6 | Graptopetalum_paraguayense vs NC_069572 |
| 0.2339 | *nad*6 | Graptopetalum_paraguayense vs NC_070303.1 |
| 0.215427 | *nad*6 | Graptopetalum_paraguayense vs NC_080974.1 |
| 0.166303 | *nad*6 | Graptopetalum_paraguayense vs NC_086673.1 |
| 0.218382 | *nad*6 | Graptopetalum_paraguayense vs NC_087799.1 |
| 0.191996 | *nad*6 | Graptopetalum_paraguayense vs PP024540.1 |
| 1.1303 | *nad*7 | Graptopetalum_paraguayense vs MW971440.1 |
| 0.765151 | *nad*7 | Graptopetalum_paraguayense vs NC_069572 |
| 1.08174 | *nad*7 | Graptopetalum_paraguayense vs NC_070303.1 |
| 0.959912 | *nad*7 | Graptopetalum_paraguayense vs NC_080974.1 |
| NA | *nad*7 | Graptopetalum_paraguayense vs NC_086673.1 |
| 0.935678 | *nad*7 | Graptopetalum_paraguayense vs NC_087799.1 |
| 1.08174 | *nad*7 | Graptopetalum_paraguayense vs PP024540.1 |
| 0.439854 | *nad*9 | Graptopetalum_paraguayense vs MW971440.1 |
| 0 | *nad*9 | Graptopetalum_paraguayense vs NC_069572 |
| 0 | *nad*9 | Graptopetalum_paraguayense vs NC_070303.1 |
| 0.364178 | *nad*9 | Graptopetalum_paraguayense vs NC_080974.1 |
| 0.356217 | *nad*9 | Graptopetalum_paraguayense vs NC_086673.1 |
| 0.373151 | *nad*9 | Graptopetalum_paraguayense vs NC_087799.1 |
| 0.269314 | *nad*9 | Graptopetalum_paraguayense vs PP024540.1 |
| NA | *rpl*10 | Graptopetalum_paraguayense vs MW971440.1 |
| 0.302174 | *rpl*10 | Graptopetalum_paraguayense vs NC_069572 |
| 0.218261 | *rpl*10 | Graptopetalum_paraguayense vs NC_070303.1 |
| NA | *rpl*10 | Graptopetalum_paraguayense vs NC_080974.1 |
| NA | *rpl*10 | Graptopetalum_paraguayense vs NC_086673.1 |
| NA | *rpl*10 | Graptopetalum_paraguayense vs NC_087799.1 |
| 0.218261 | *rpl*10 | Graptopetalum_paraguayense vs PP024540.1 |
| NA | *rpl*16 | Graptopetalum_paraguayense vs MW971440.1 |
| 0.519517 | *rpl*16 | Graptopetalum_paraguayense vs NC_069572 |
| NA | *rpl*16 | Graptopetalum_paraguayense vs NC_070303.1 |
| 0.310783 | *rpl*16 | Graptopetalum_paraguayense vs NC_080974.1 |
| NA | *rpl*16 | Graptopetalum_paraguayense vs NC_086673.1 |
| 0.184862 | *rpl*16 | Graptopetalum_paraguayense vs NC_087799.1 |
| 0.0705256 | *rpl*16 | Graptopetalum_paraguayense vs PP024540.1 |
| NA | *rpl*5 | Graptopetalum_paraguayense vs MW971440.1 |
| NA | *rpl*5 | Graptopetalum_paraguayense vs NC_069572 |
| 0.219569 | *rpl*5 | Graptopetalum_paraguayense vs NC_070303.1 |
| 0.314193 | *rpl*5 | Graptopetalum_paraguayense vs NC_080974.1 |
| 0.387194 | *rpl*5 | Graptopetalum_paraguayense vs NC_086673.1 |
| 0.361117 | *rpl*5 | Graptopetalum_paraguayense vs NC_087799.1 |
| 0.219569 | *rpl*5 | Graptopetalum_paraguayense vs PP024540.1 |
| 0.395194 | *rps*12 | Graptopetalum_paraguayense vs MW971440.1 |
| 0.252658 | *rps*12 | Graptopetalum_paraguayense vs NC_069572 |
| NA | *rps*12 | Graptopetalum_paraguayense vs NC_070303.1 |
| 0.315465 | *rps*12 | Graptopetalum_paraguayense vs NC_080974.1 |
| NA | *rps*12 | Graptopetalum_paraguayense vs NC_086673.1 |
| 0.263135 | *rps*12 | Graptopetalum_paraguayense vs NC_087799.1 |
| 0 | *rps*12 | Graptopetalum_paraguayense vs PP024540.1 |
| NA | *rps*13 | Graptopetalum_paraguayense vs MW971440.1 |
| 0.291427 | *rps*13 | Graptopetalum_paraguayense vs NC_069572 |
| 0 | *rps*13 | Graptopetalum_paraguayense vs NC_070303.1 |
| 0.449585 | *rps*13 | Graptopetalum_paraguayense vs NC_080974.1 |
| NA | *rps*13 | Graptopetalum_paraguayense vs NC_086673.1 |
| 0.447108 | *rps*13 | Graptopetalum_paraguayense vs NC_087799.1 |
| 0 | *rps*13 | Graptopetalum_paraguayense vs PP024540.1 |
| 0.511156 | *rps*7 | Graptopetalum_paraguayense vs MW971440.1 |
| NA | *rps*7 | Graptopetalum_paraguayense vs NC_069572 |
| NA | *rps*7 | Graptopetalum_paraguayense vs NC_070303.1 |
| 0.464736 | *rps*7 | Graptopetalum_paraguayense vs NC_080974.1 |
| 0.355254 | *rps*7 | Graptopetalum_paraguayense vs NC_086673.1 |
| 0.397489 | *rps*7 | Graptopetalum_paraguayense vs NC_087799.1 |
| 0.0964007 | *rps*7 | Graptopetalum_paraguayense vs PP024540.1 |

Note: Vs represents the computational results between species sample1 and sample2.

Table S10. Mitochondrial Sequence Collinearity Analysis Results.

| #ref | ref len | ref homo len | prop in ref | query | query len | query homo len | prop in query |
| --- | --- | --- | --- | --- | --- | --- | --- |
| *Graptopetalum paraguayense* | 242059 | 63811 | 26.36% | *Tetragonia tetragonoides* | 347227 | 65102 | 18.75% |
| *Graptopetalum paraguayense* | 242059 | 138646 | 57.28% | *Sedum plumbizincicola* | 212159 | 137236 | 64.69% |
| *Graptopetalum paraguayense* | 242059 | 88955 | 36.75% | *Rhodiola crenulata* | 194106 | 94002 | 48.43% |
| *Graptopetalum paraguayense* | 242059 | 66957 | 27.66% | *Mammillaria huitzilopochtli* | 2052004 | 67745 | 3.30% |
| *Graptopetalum paraguayense* | 242059 | 25949 | 10.72% | *Mesembryanthemum crystallinum* | 1005707 | 29523 | 2.94% |
| *Graptopetalum paraguayense* | 242059 | 66862 | 27.62% | *Nopalea cochenillifera* | 1156235 | 77892 | 6.74% |
| *Graptopetalum paraguayense* | 242059 | 103776 | 42.87% | *Rhodiola rosea* | 259150 | 130025 | 50.17% |

Table S11. The Pi value of each gene.

| #No. | Region | Pi | Total Number of mutations | Region length |
| --- | --- | --- | --- | --- |
| 1 | *atp*1 | 0.04396 | 132 | 1533 |
| 2 | *atp*4 | 0.03589 | 43 | 597 |
| 3 | *atp*6 | 0.0265 | 36 | 897 |
| 4 | *atp*8 | 0.08645 | 97 | 495 |
| 5 | *atp*9 | 0.15809 | 66 | 510 |
| 6 | *ccm*B | 0.0364 | 51 | 621 |
| 7 | *ccm*C | 0.05701 | 87 | 753 |
| 8 | *ccm*Fc | 0.05425 | 137 | 1377 |
| 9 | *ccm*Fn | 0.06365 | 194 | 1740 |
| 10 | *cob* | 0.04108 | 109 | 1200 |
| 11 | *cox*1 | 0.03683 | 112 | 1584 |
| 12 | *cox*2 | 0.0662 | 110 | 845 |
| 13 | *cox*3 | 0.04534 | 81 | 798 |
| 14 | *mat*R | 0.04386 | 112 | 2071 |
| 15 | *mtt*B | 0.0534 | 43 | 828 |
| 16 | *nad*1 | 0.02721 | 50 | 978 |
| 17 | *nad*2 | 0.01988 | 46 | 1470 |
| 18 | *nad*3 | 0.0381 | 25 | 357 |
| 19 | *nad*4 | 0.04099 | 155 | 1488 |
| 20 | *nad*4L | 0.03 | 17 | 303 |
| 21 | *nad*5 | 0.03104 | 162 | 2016 |
| 22 | *nad*6 | 0.05369 | 72 | 965 |
| 23 | *nad*7 | 0.01204 | 27 | 1185 |
| 24 | *nad*9 | 0.0409 | 44 | 579 |
| 25 | *rpl*10 | 0.00818 | 7 | 489 |
| 26 | *rpl*16 | 0.03724 | 33 | 516 |
| 27 | *rpl*5 | 0.07741 | 114 | 563 |
| 28 | *rps*12 | 0.02945 | 22 | 378 |
| 29 | *rps*13 | 0.05299 | 38 | 351 |
| 30 | *rps*7 | 0.06349 | 65 | 447 |
| 31 | *rrn*18 | 0.0258 | 89 | 2212 |
| 32 | *rrn*26 | 0.01596 | 47 | 3587 |
| 33 | *rrn*5 | 0.02663 | 7 | 121 |

Note: "Total Number of mutations" indicates the count of variant sites, and "Region length" represents the aligned gene length.
